# Aquaporin 1 overexpression may enhance glioma tumorigenesis by interacting with the transcriptional regulation networks of Foxo4, Maz, and E2F families

**DOI:** 10.1186/s41016-023-00342-3

**Published:** 2023-12-06

**Authors:** Ying Guan, Jinhua Han, Die Chen, Yuefu Zhan, Jianqiang Chen

**Affiliations:** 1 Department of Ultrasound, The First Affiliated Hospital of Hainan Medical College, Haikou City, 570102 Hainan Province China; 2 Department of Radiology, The First Affiliated Hospital of Hainan Medical College, Haikou City, 570102 Hainan Province China; 3 Department of Radiology, Hainan Children’s Hospital, Haikou City, 571103 Hainan Province China

**Keywords:** C6 cell line, Aquaporin 1, Gioma, Migration

## Abstract

**Background:**

The glioblastoma has served as a valuable experimental model system for investigating the growth and invasive properties of glioblastoma. Aquaporin-1 (AQP1) in facilitating cell migration and potentially contributing to tumor progression. In this study, we analyzed the role of AQP1 overexpression in glioblastoma and elucidated the main mechanisms involved.

**Methods:**

AQP1 overexpression recombinant vector was introduced into C6 rat glioma cells to construct an AQP1 overexpression C6 cell line, and its effect on cell viability and migration ability was detected by MTT and Transwell. RNA was extracted by Trizol method for gene sequencing and transcriptomics analysis, and the differentially expressed genes (DEGs) were enriched for up- and downregulated genes by Principal component analysis (PCA), and the molecular mechanism of AQP1 overexpression was analyzed in comparison with the control group using the NCBI GEO database. Statistical analysis was performed using Mann-Whitney paired two tailed *t* test.

**Results:**

The cell viability of AQP1-transfected cell lines increased by 23% and the mean distance traveled increased by 67% compared with the control group. Quantitative analysis of gene expression showed that there were 12,121 genes with an average transcripts per million (TPM) value greater than 1. DEGs accounted for 13% of the genes expressed, with the highest correlation with upregulated genes being FOXO4 and MAZ, and the highest with downregulated genes being E2F TFs.

**Conclusions:**

AQP1 may be implicated in glioma formation by interacting with the transcriptional regulation networks involving the FOXO4, MAZ, and E2F1/2. These findings shed light on the potential significance of AQP1 in glioma pathogenesis and warrant further investigations to unravel the underlying molecular mechanisms.

**Supplementary Information:**

The online version contains supplementary material available at 10.1186/s41016-023-00342-3.

## Background

Malignant gliomas, the most prevalent primary intracranial tumors in adults, are associated with unfavorable therapeutic outcomes and high mortality rates [1, 2]. Despite advancements in therapeutic methods and strategies, the average survival time for patients with gliomas following treatment remains less than 15 months due to significant disease progression and recurrence [3]. Gliomas exhibit rapid growth, active microvascular proliferation, heightened glucose consumption, intratumoral necrosis, and hypoxia, as well as vasogenic brain edema [4, 5]. The aggressive and recurring nature of gliomas has been attributed to diffuse invasion, microvascular proliferation, and strong resistance to apoptosis [6]. A characteristic common to malignant gliomas is the occurrence of brain edema, which has been linked to the functioning of Aquaporin 1 (AQP1) [7].

AQP1 is a highly conserved gene that encodes a transmembrane channel protein responsible for facilitating water transport across hydrophobic cell membranes [8]. Emerging evidence suggests that AQP1 possesses multiple additional functions beyond water transportation. For instance, the expression of AQP1 has been found to correlate with the malignancy grade of gliomas [9, 10] as well as the treatment outcomes of various malignant cancer [11–13]. Increased AQP1 expression has been shown to enhance the invasive capabilities of glioma cells [14, 15]. Studies conducted on AQP1 knockout (KO) mice have demonstrated impaired tumor growth, cell migration, and angiogenesis [16, 17]. Similarly, dexamethasone, a commonly used medication for glioma treatment, inhibits C6 cell proliferation mediated by AQP1 [18].

To investigate the role of AQP1 in glioma formation, we conducted an experiment where we overexpressed AQP1 in the rat C6 glioma cell line. The C6 cell line has undergone extensive in vitro culturing for numerous passages, and its tumorigenicity has remained relatively stable. While the precise composition of the C6 glioma cell line in terms of cancer stem cells remains a topic of debate with varying reported percentages [19], we observed that the overexpression of AQP1 led to increased cell viability and cell migration. Through transcriptomic analysis, we identified that AQP1 overexpression may enhance glioma tumorigenesis by influencing the transcriptional regulation networks involving Foxo4, Maz, and E2F families.

## Methods

### Cell culture and transfection

C6 rat glioma cells were purchased from the Icell Bioscience company. The cells were cultured in Dulbecco’s modified Eagle medium (DMEM) containing 10% FBS, 100 U/ml penicillin and 100 µg/ml streptomycin (PenStrep) (GIBCO, Invitrogen, Paisley, Scotland, UK). Cell cultures were maintained at 37 °C in a humidified chamber of 95% air and 5% CO_2_. The AQP1 sequence was amplified using high-fidelity polymerase and ligated into the expression vector pCDH-CMV-MCS-EF1-CopGFP-T2A-puro (pCDh). Then, the recombinant vector was introduced into C6 cells and the insert sequence was validated by Sanger sequencing.

### Western blotting

Cells were lyzed in RIPA buffer (50 mM Tris-HCl, pH 7.5, 0.1% sodium deoxycholate, 1% Nonidet P-40, 0.1% SDS, 150 mM NaCl) supplied with protease inhibitors (Roche, Penzberg, Upper Bavaria, Germany). The protein concentration was examined by Bradford methods (Bio-Rad). The proteins were separated by SDS-PAGE gels and transferred onto PVDF membranes (Millipore, Bedford, USA). Then the membranes were sequentially incubated with indicated primary antibodies and HRP-coupled secondary antibodies. Protein bands were visualized and detected with the enhanced chemiluminescence Detection System (ThermoFisher, USA).

### MTT cell viability assay

MTT was used for detecting the cell viability. After cells were cultured for 24 h, 20 μl 5 mg/mL MTT was administrated to each well. Cells were cultured for 4 h. Afterward, we used 150 μl dimethyl sulfoxide to lyse formazan crystal. The value was obtained at 570 nm by a multiwell spectrophotometer.

### Transwell migration assay

To perform transwell migration assay, 5 × 10^4^ cells in 500 μl per chamber FBS-free medium were seeded to the upper chamber of uncoated 24-well chambers/microfilters (BD, Franklin Lakes, NJ, USA). The lower chamber was added with medium supplemented with 10% FBS. Cell motility/migration was determined according to the number of cells migrated through micropores in a certain area of the microfilter in 24 h.

### RNA extraction

Total RNA was extracted using Trizol (Invitrogene, Lot# 15596-018) and genomic DNA contamination removed with RNase-free DNase I (Transgen, Code#GD201-01) following the manufacturer’s protocols. RNA degradation and contamination were monitored on 1% agarose gels. RNA was reverse-transcribed into cDNA using the M-MLV Reverse Transcriptase (Promega, Lot# M5301).

### RNA sequencing and analysis

The library construction and Illumina sequencing were performed at WuXi NextCODE, Wuxi, China (www.wuxinextcode.com). Then, paired-end reads were sequenced using the Illumina HiSeq platform. The sequencing quality was examined using FastQC software (v0.11.2). Reads containing adapter, reads containing ploy-N and low-quality reads from raw reads were removed to obtain filtered reads with Trimmomatic (v0.36) [20]. All the downstream analyses were based on the filtered reads with high quality and at least 75 bp. The paired-end filtered reads were aligned to the rat reference genome (rn6) using STAR (v2.5.1b) [21]. The gene expression quantification was conducted using RSEM (v1.2.29) [22]. Only genes with average TPM (Transcripts Per Million) value greater than 1 in at least one group of samples were considered expressed and used for the differential expression analysis. Differentially expressed genes (DEGs), defined as genes with fold change (FC) > 2 and FDR < 0.05, were identified using DESeq2 package (v 1.14.1) [23]. The functional enrichment analysis of DEGs was done using ToppGene Suite [24]. ToppGene terms with corrected P value (Benjamini) less than 10^−5^ were considered significant. The RNA-seq data was deposited into the Gene Expression Omnibus (GEO) database (accession number: GSE137733).

### Real-time quantitative PCR (RT-qPCR)

Total RNA was purified with TRIzol (Invitrogen) according to the manufacture’s instruction. To convert RNA into cDNA, Mixed 1 µg RNA and 1 µg Oligo dT in a final volume of 10 µl, and then heated the mixture at 70 °C for 5 min, followed by cooling on ice. The cooled mixture was added 5 µl reaction buffer, 1.25 µl dNTPs mix (each 10 µM), 1 µl 200 U/µl reverses transcript enzyme and 1 µl 40 U/µl RNase inhibitor in a final 20 µl volume, and then incubated at 42 °C for 1 h. All reagents used in the reverse transcription assay were bought from Promega. Real-time PCR was carried out using SYBR Green II Master Mix (TaKaRa, Japan) with Applied Biosystems 7300 Real-Time PCR System using the following gene specific primers, AQP1, 5′-CTGTGGTGGCTGAGTTCCTG-3′ and 5′-ACCTCGGCCAAGTGAGTTCTC-3′; VEGF, 5′-CAAACCTCACCAAAGCCAGC-3′ and 5′-ACGCGAGTCTGTGTTTTTGC-3′; GAPDH as the internal control, 5′-ACCACAGTCCATGCCATCAC-3′ and 5′-TCCACCACCCTGTTGCTGTA-3′.

### Data analysis

Statistical analysis was performed using Mann-Whitney paired two tailed *t* test (GraphPad Prism v5.0). The qRT-PCR data was analyzed with Quanti v1.3.1 Software (Thermo Scientific, USA). The threshold cycle (CT) cut off based on the negative and the positive controls. Tests were considered statistically significant when *P* values were < 0.05.

## Results

### Overexpression of AQP1 in rat C6 glioma cell line

**Fig. 1:**
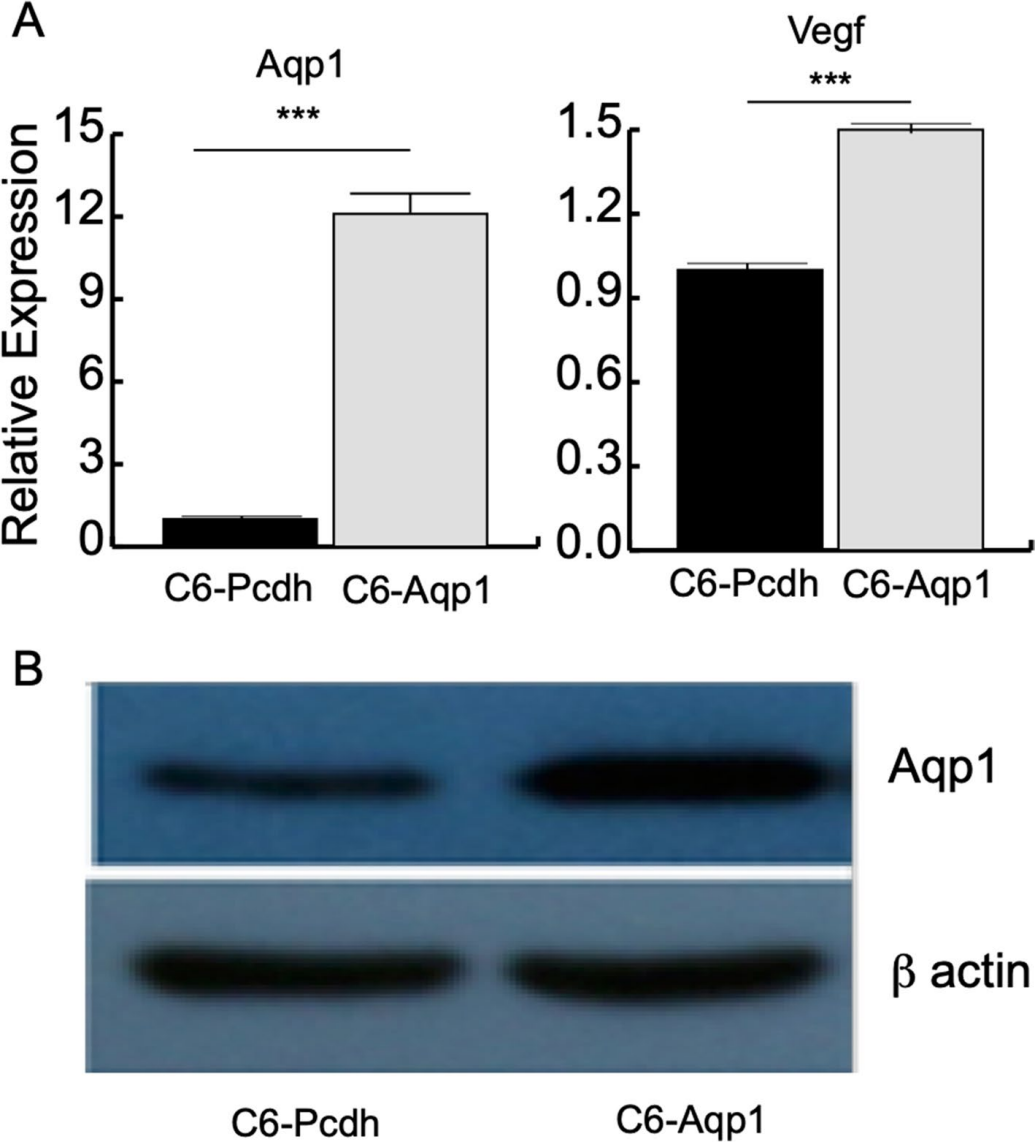
Overexpression of AQP1 in rat C6 glioma cell line. **A** Relative gene expression of Aqp1 and Vegf in C6-Pcdh and C6-AQP1 cell strains compared to Gapdh by quantitative real-time PCR for AQP1 and Vegf (*n* = 3, *t* test, ***p value < 0.001). **B** Western blot of Aqp1 protein in C6-Pcdh and C6-Aqp1 cell strains

To investigate the potential modulatory effect of AQP1 overexpression on gene expression in C6 cells, we transfected the C6 cell line with AQP1 expression plasmids. Subsequently, we performed a comparative analysis of AQP1 gene expression by RT-qPCR, normalized against the housekeeping gene GAPDH, in two cell lines: the AQP1-transfected cell line (C6-AQP1) and the control cell line (C6-Pcdh). In the C6-Pcdh cell line, the average change in AQP1 expression was approximately 1-fold. In contrast, the C6-AQP1 cell line exhibited a substantial increase in AQP1 gene expression, with an average fold change of 11.1, which was significantly higher than the control (Fig. 1A). Considering that AQP1 overexpression has been reported to enhance Vegf expression [25], we also examined the expression level of Vegf in both C6-Pcdh and C6-AQP1 cell lines. As anticipated, the expression level of Vegf remained relatively unchanged in C6-Pcdh, while it showed a 1.5-fold increase in C6-AQP1 (Fig. 1A). These results suggest that the transfection of AQP1 was successful and elicited the expected molecular responses in the cell lines. To further confirm the overexpression of AQP1 in the C6 cell line, we examined the protein expression level using western blotting. The protein levels of beta actin were similar between C6-Pcdh and C6-AQP1, while that of AQP1 was higher in C6-AQP1 than in C6-Pcdh (Fig. 1B). Taken together, the RT-qPCR and western blotting experiments support that we succeed in overexpressing AQP1 in the C6 cell line.

### Increased cell viability and cell migration after AQP1 transfection

**Fig. 2:**
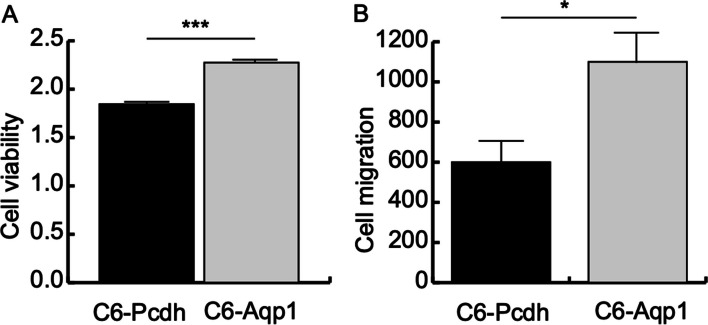
Phenotypic changes of C6 cells after AQP1 transfection. **A** Increased cell viability (*n* = 3, *t* test, ***p value < 0.001). **B** Increased cell migration (*n* = 3, *t* test, ***p value < 0.001)

AQP subtypes, such as AQP1, AQP3, and AQP5, have been implicated in various aspects of carcinogenesis, tumor progression, and invasion [26]. To investigate the impact of AQP1 overexpression on cell growth and migration in C6 cells, we conducted MTT cell viability assays and transwell migration assays. The MTT assay revealed that both Pcdh and AQP1 overexpression in C6 cells resulted in increased cell viability. However, the increase in cell viability was 23% higher in the cell strains transfected with AQP1 compared to those transfected with Pcdh (Fig. 2A). Furthermore, the transwell migration assay demonstrated that C6 cells transfected with Pcdh exhibited an average migration distance of 600 μm within 24 h, while cells transfected with AQP1 displayed an average migration distance of 1000 μm within the same timeframe, representing a 67% increase compared to the control (Fig. 2B). Taken together, these findings from the MTT cell viability assay and transwell migration assay provide compelling evidence that the overexpression of AQP1 in C6 cells significantly enhances both cell viability and migration capabilities.

### Transcriptomic analysis and evaluation

To investigate the genome-wide effects of AQP1 overexpression in C6 cells, we performed transcriptome analysis using RNA-seq. We obtained an average of 119,808,886 and 107,152,564 raw reads for the C6 and C6-AQP1 samples, respectively, with 96.4% and 96.2% of the reads successfully mapped to the rat genome. Gene expression quantification revealed that out of the 32,754 genes examined, 12,121 genes exhibited an average transcripts per million (TPM) value greater than 1, and subsequent analyses were conducted based on these expressed genes.

**Fig. 3:**
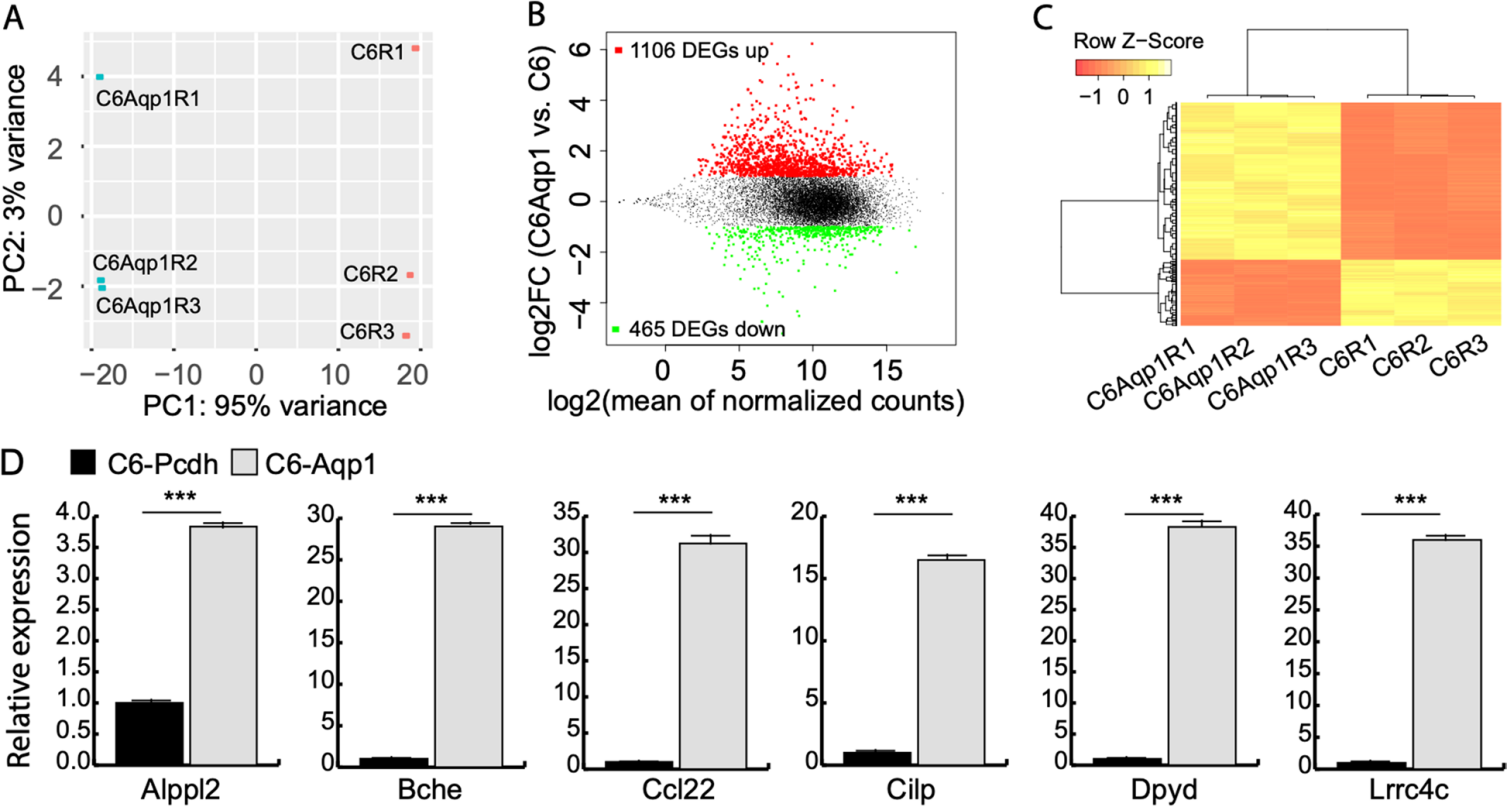
Identification of DEGs from RNA-seq data. **A** PCA analysis of RNA-seq samples based on the gene expression profiles of 12,121 genes using the plotPCA function in DESeq2 package. **B** MA plot of RNA-seq data. Red points stand for upregulated DEGs and green points for down-regulated DEGs. **C** Heatmap of DEGs drawn using the heatmap.2 function in the R package gplots. **D** RT-qPCR validation of DEGs (*n* = 3, *t* test, ***p value < 0.001)

Principal component analysis (PCA) was performed to assess the variation among the samples. The results demonstrated that 95% of the variation observed among the samples could be attributed to PC1, which represents the difference before and after AQP1 overexpression in C6 cells (Fig. 3A). This finding indicates significant differences in gene expression profiles between the pre- and post-transfection stages of AQP1 in C6 cells, as captured by the PCA.

A total of 1571 differentially expressed genes (DEGs), accounting for 13% of the expressed genes, were identified following AQP1 transfection in C6 cells (Fig. 3B, Additional file 1: Table S1). Among these DEGs, 1106 genes were upregulated, while 465 genes were downregulated. These findings align with our previous RT-qPCR results (Fig. 1A) and demonstrate a significant increase in the normalized read count of AQP1 from 12,371.43 to 37,568.23 (log2 fold change = 1.6, FDR = 0, Fig. 3B, Additional file 1: Table S1) after AQP1 transfection. This further validates the successful overexpression of Aqp1 in C6 cells. The overall expression pattern of the DEGs indicates that the changes induced by AQP1 expression in C6 cells were both substantial and consistent (Fig. 3C).

To validate the results obtained from RNA-seq, we randomly selected six DEGs and assessed their expression using RT-qPCR. The results demonstrated significant changes in all six genes after AQP1 overexpression (Fig. 3D), consistent with the findings from the RNA-seq analysis.

### AQP1 may enhance tumor progression by interacting with FOXO4, MAZ, and E2F TF families

To gain insights into the functional implications of the differentially expressed genes (DEGs), we performed gene enrichment analysis using the ToppGene suite [24]. Separate analyses were conducted for the upregulated and downregulated DEGs (Additional file 2: Table S2).

**Fig. 4:**
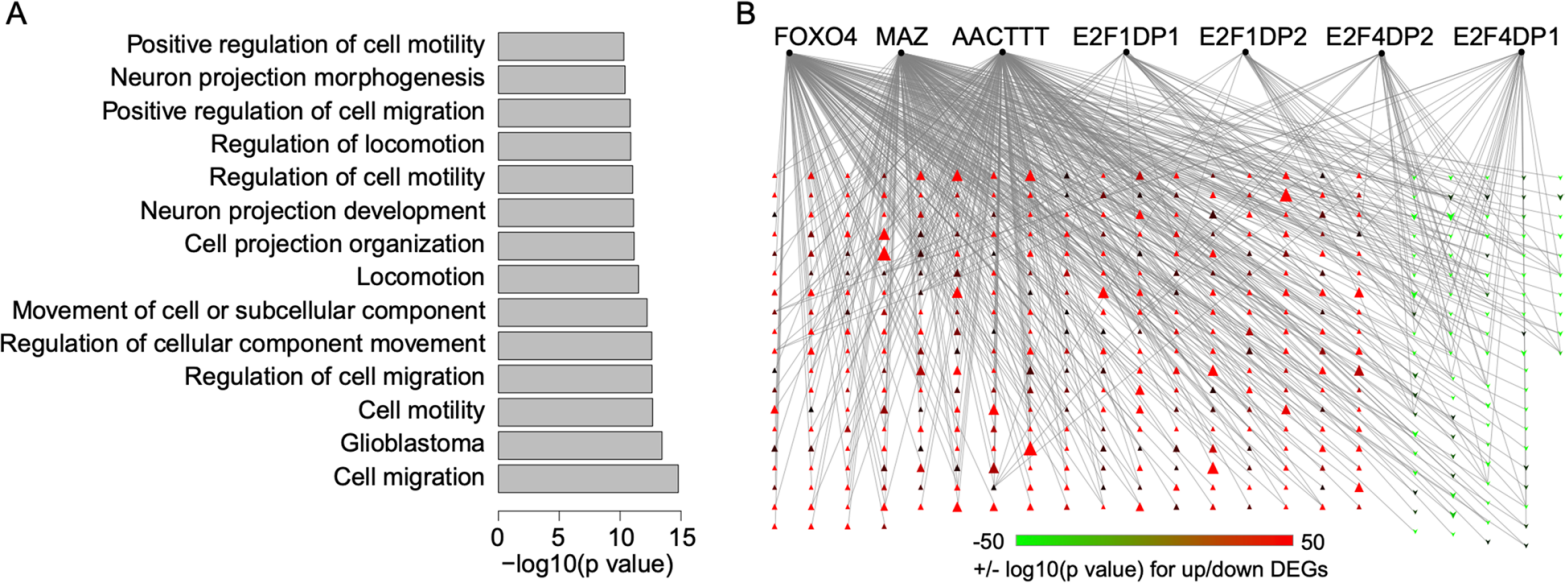
Functional analysis of DEGs. **A** Enriched functional terms related to glioma and cell mobility. **B** Potential transcriptional regulation of DEGs by transcriptional factors. The black dots represent transcriptional factors except for AACTT, which is the binding motif for an unknown TF. The triangles represent upregulated (red) and downregulated (green) DEGs. The lines between TFs and DEGs mean there are binding sites of the TFs for DEGs. The network was generated using Cytoscape (v 3.7.1) and edited using Adobe Illustrator

Among the enrichment results for the upregulated DEGs, we observed that glioblastoma ranked as the top disease in the disease category (Fig. 4A, Additional file 2: Table S2), suggesting the overexpression of AQP1 in the C6 cell line strengthened the expression of genes associated with glioblastoma. Additionally, we identified significant enrichment of Gene Ontology (GO) terms related to cell migration and motility as top functional categories (Fig. 4A), which aligns with the observed increase in cell migration following AQP1 overexpression in C6 cells (Fig. 2B). For the downregulated genes, the top functions affected included cell cycle, mRNA catabolic process, organic cyclic compound catabolic process, and macromolecule catabolic process (Additional file 2: Table S2).

Furthermore, the analysis of transcription factor (TF) binding sites revealed that the top TFs associated with the upregulated DEGs were FOXO4, a TF with a complex role in cancer that may contribute to cancer progression [27]; MAZ, which has been implicated in promoting pancreatic cancer cell invasion via CRAF–ERK signaling [28]; and an unidentified TF with the binding motif “AACTTT”. As for the downregulated DEGs, the top TFs were E2F4/DP1, E2F1/DP2, E2F4/DP2, and E2F1/DP1 (Fig. 4B, Additional file 2: Table S2), which are members of the E2F transcription factor family known to be involved in cell cycle control and cancer progression [29].

In summary, these results suggest that AQP1 overexpression may enhance tumor progression by interacting with the transcriptional regulation networks governed by FOXO4, MAZ, and E2F TFs.

## Discussion

In this study, we aimed to investigate the role of AQP1 in glioma formation by overexpressing it in the C6 glioma cell line. Initially identified as a water channel protein [30], AQP1 has also been implicated in cell migration and tumor formation [25]. Consistent with previous findings, our research demonstrated that overexpression of AQP1 in C6 cells significantly increased cell migration (Fig. 2B) [31–33]. Notably, the gene expression profile revealed a significant upregulation of genes associated with cell migration following AQP1 overexpression (Fig. 4A), which is consistent with the observed phenotype (Fig. 2B). The enhanced mobility of C6 cells after AQP1 overexpression suggests a transformation from cancer stem cells, which constitute a major portion of the C6 glioma cell line [19], into invasive cancer cells [34]. Intriguingly, the upregulated genes after AQP1 overexpression were strongly associated with glioblastoma, as indicated by the top disease ranking (Fig. 4A, Additional file 2: Table S2), suggesting a potential involvement of AQP1 in tumorigenesis.

In our comprehensive analysis using the ToppGene Suite (Additional file 2: Table S2–3), we discovered that the up- and downregulated genes shared binding sites for specific transcription factors known to be involved in cancer progression. Among the upregulated DEGs, FOXO4 (Fig. 4B, Additional file 2: Table S2), a member of the forkhead family transcription factors O subclass, was prominently shared. Depending on its modification state, FOXO4 can regulate various cellular pathways, including oxidative stress signaling, longevity, insulin signaling, cell cycle progression, and apoptosis [35]. While FOXO transcription factors have traditionally been considered tumor suppressors due to their inhibitory effect on cancer cell growth and survival, they can also promote tumor development and progression by maintaining cellular homeostasis, facilitating metastasis, and inducing therapy resistance [27]. Another shared transcription factor among the upregulated DEGs is MYC-associated zinc finger protein (MAZ) (Fig. 4B, Additional file 2: Table S2), which has been implicated in promoting pancreatic cancer cell invasion [28]and hepatocellular carcinoma metastasis through the induction of epithelial-mesenchymal transition [36]. Among the downregulated DEGs, the shared transcription factors include E2F4/DP1, E2F1/DP2, E2F4/DP2, and E2F1/DP1 (Fig. 4B, Additional file 2: Table S3), which have been identified as important contributors to tumor progression in various cancer types [29, 37]. These transcription factors associated with the DEGs suggest that AQP1 overexpression may interact with their transcriptional regulatory networks, ultimately promoting C6 cell viability and migration.

## Conclusions

In conclusion, our study demonstrated significant phenotypic and transcriptomic changes upon overexpression of AQP1 in C6 cell lines. AQP1 overexpression resulted in increased cell viability and cell migration, indicating its functional impact on glioma progression. Through comprehensive transcriptome analysis, we identified extensive changes associated with key biological processes such as cell migration, glioblastoma, and neuron projection morphogenesis. Moreover, transcription factor binding analysis revealed that the upregulated genes were likely regulated by Foxo4 and Maz, while the downregulated genes exhibited binding sites for E2F1 and E2F2. These findings suggest that AQP1 overexpression may enhance glioma tumorigenesis by interacting with the transcriptional regulation networks involving Foxo4, Maz, and E2F families. The data presented in this study shed light on the potential involvement of AQP1 in glioma progression and provide insights into the dysregulation of gene expression in glioma. These discoveries may offer valuable clues for developing strategies to intervene in gene expression and potentially contribute to the treatment of glioma.

### Supplementary Information

**Additional file 1.** 

**Additional file 2.** 

## Data Availability

The datasets generated during the current study are available in the GEO database (GSE137733).

## Abbreviations

AQP1: Aquaporin-1
DEGs: Differentially expressed genes
GO: Gene ontology
PCA: Principle component analysis
RT-qPCR: Real-time quantitative PCR
